# Supplementary material for: A positive correlation between GC content and growth temperature in prokaryotes
Source: BMC Genomics. 2022 Feb 9;23:110. doi: 10.1186/s12864-022-08353-7 (PMC8827189; doi:10.1186/s12864-022-08353-7)
Supplement: Supplementary file 3 — Additional file 3: Fig. S1. Nonlinearity in the relationship between prokaryotic optimal growth temperature and GC contents. It was estimated using the generalized additive mixed model (GAMM) by adjusting for the genus as a random effect. The dataset including 681 bacterial and 155 archaeal species was used in this analysis. The effective degrees of freedom (edf) is a proxy for the level of nonlinearity in the relationships. We presented the relationships of optimal growth temperature with the GC contents of protein-coding sequences and non-coding DNA (intergenic sequences and untranslated regions of mRNA that are generally unannotated in prokaryotic genomes) as (A) and (B) in this figure. The significance values of the results presented in (A) and (B) are P = 5 × 10^−5^ and 9 × 10^−4^, respectively. [file 12864_2022_8353_MOESM3_ESM.docx]

**A positive correlation between GC content and growth temperature in prokaryotes**

En-Ze Hu^†^, Xin-Ran Lan^†^, Zhi-Ling Liu, Jie Gao, and Deng-Ke Niu*

MOE Key Laboratory for Biodiversity Science and Ecological Engineering and Beijing Key Laboratory of Gene Resource and Molecular Development, College of Life Sciences, Beijing Normal University, Beijing, 100875, China

^†^These authors contributed equally to this paper.

*Corresponding author: Deng-Ke Niu, [dkniu@bnu.edu.cn](mailto:dkniu@bnu.edu.cn), [dengkeniu@hotmail.com](mailto:dengkeniu@hotmail.com)





Fig. S1. Nonlinearity in the relationship between prokaryotic optimal growth temperature and GC contents. It was estimated using the generalized additive mixed model (GAMM) by adjusting for the genus as a random effect. The dataset including 681 bacterial and 155 archaeal species was used in this analysis. The effective degrees of freedom (edf) is a proxy for the level of nonlinearity in the relationships. We presented the relationships of optimal growth temperature with the GC contents of protein-coding sequences and non-coding DNA (intergenic sequences and untranslated regions of mRNA that are generally unannotated in prokaryotic genomes) as (A) and (B) in this figure. The significance values of the results presented in (A) and (B) are *P* = 5 × 10^−5^ and 9 × 10^−4^, respectively.
